# Beyond Women's Health: Long-Term Human Papillomavirus–Related Cancer Trends in Norway

**DOI:** 10.1093/infdis/jiaf351

**Published:** 2025-07-09

**Authors:** Thea E Hetland Falkenthal, Ståle Nygård, Mari Nygård

**Affiliations:** Department of Research, Cancer Registry of Norway, Norwegian Institute of Public Health, Oslo, Norway; Department of Research, Cancer Registry of Norway, Norwegian Institute of Public Health, Oslo, Norway; Department of Research, Cancer Registry of Norway, Norwegian Institute of Public Health, Oslo, Norway

**Keywords:** Human Papilloma virus, Cancer, Cervical cancer, oropharyngeal cancer

## Abstract

**Background:**

Understanding the total burden of human papillomavirus (HPV)–related cancers is crucial for improving prevention strategies. While organized cervical cancer (CC) screening has been implemented for many years, other HPV-related cancers lacked screening programs. Primary prevention through HPV vaccination has been implemented through national programs, initially for girls and later for boys. To analyze changes in HPV-attributable cancer incidence for both men and women, we used data from the Cancer Registry of Norway (CRN).

**Methods:**

This was an observational population-based study using high-quality data from the CRN. The proportion of cancers at each site attributed to HPV was calculated based on existing literature. We estimated HPV cancer incidence rates from 1990 to 2023 and forecasted incidences until 2038 for cervical squamous cell carcinoma (SCC) and adenocarcinoma, along with other HPV-related SCCs.

**Results:**

Among men, HPV-attributable cancer incidence was rising, with male oropharyngeal SCC showing the fastest increase (annual percentage change [APC], 4.5; *P* < .01). Overall, the incidence of HPV-attributable cancers not prevented by screening steadily increased (APC, 2.8; *P* < .01), surpassing CC incidence and projected to continue rising until 2038. In women, CC remains the most common HPV cancer. However, after an increasing trend since 2004, cervical SCC incidence rates decreased 6% annually from 2018 to 2023 (95% confidence interval [CI], −9.9 to −1.9; *P* < .01).

**Conclusions:**

The burden of HPV-related cancers beyond CC is increasing in Norway, whereas CC incidence is declining. Addressing the rising total burden of HPV-attributable cancers requires additional preventive measures.

Human papillomavirus (HPV) is a common sexually transmitted infection (STI). There are >100 types of HPV, and 13 of these are considered high-risk (HR-HPV) types for which persistent infection may lead to precancer and cancer [1]. The most frequent cancer caused by HPV is cervical cancer (CC), with all squamous cell carcinomas (SCCs) and most adenocarcinomas (ACs) being caused by HR-HPV infection [1, 2]. A considerable proportion of oropharyngeal and anal cancer in both sexes, penile cancer in men, and vulvar and vaginal cancers in women are also caused by HR-HPV [3].

In this study, we define “HPV-related cancers” as cancer types for which HPV is a known cause, irrespective of the proportion actually attributed to HPV in a given population. In contrast, “HPV-attributable cancers” refers to those cancers actually caused by HPV.

Globally, the time trends of HPV-related cancers at noncervical sites are difficult to document [4], and the proportion of these cancers attributed to HPV varies between geographical regions. The highest increase in cancer burden attributed to HPV is observed in high-income countries [1]. In Nordic countries, where high-accuracy cancer registries are available, increasing long-term incidence trends for HPV-attributable cancers have been reported [5–7] and there are recent studies on the HPV-attributable fraction (HPV-AF) in several noncervical cancers [8–11].

The widespread use of cervical screening began in the 1970s, with many countries adopting organized cervical screening programs. In Norway, such a program was implemented in 1995. Through screening, precancers are detected and treated before developing into CC [12]. In recent years, HPV testing has increasingly replaced cytology as the primary screening method in many countries [13]. Initially introduced in 2015 for restricted age groups [14], HPV testing became the primary screening method in Norway by 2023. This change has led to increased detection of cervical intraepithelial neoplasia grade 2+ and is anticipated to result in a future decrease in CC [14, 15]. As of 2025, no early detection and treatment programs have been implemented for other HPV-related precancers or cancers.

In countries with high vaccination coverage, HPV-attributable cancers are expected to become rare when the vaccinated cohorts reach cancer ages. In Norway, prophylactic HPV vaccination was implemented in the national childhood immunization program for girls in 2009 and for boys in 2018 [16]. Additionally, there was a 3-year vaccine campaign for girls aged 19–26 years in 2016–2019, but no catch-up program has been offered to men.

The objective of this study is to investigate trends in HPV-attributable cancers in men and women in Norway over the last 33 years, including the introduction of HPV vaccines. We also provide forecasted incidence rates until 2038.

## METHODS

### Registry Data

Cancer data were extracted from the Cancer Registry of Norway (CRN). It is legally required to notify cancer diagnosis, and virtually all cancers diagnosed in the Norwegian population have been registered by the CRN since 1952. The Cause of Death Registry, pathology laboratories, and clinicians are the main data sources, ensuring high levels of completeness and quality of CRN data [17].

In the present study, we focus on cancers associated with HPV [1]. Thus, for the period 1990–2023, we extracted data on all incident primary cases of malignant cancer (except basal cell carcinomas) that occurred at the following anatomical sites (*International Classification of Diseases, Tenth Revision* topography code): base of tongue (C01), tonsils (C09), oropharynx (C10), anus (C21), vulva (C51), vagina (C52), cervix (C53), and penis (C60), giving a total of 22 702 incident cases. Base of tongue, tonsils, and oropharynx were grouped together in the analyses and are henceforth referred to as “oropharynx.” For each case, the registry data extracted were tumor topography and morphology, year of diagnosis, and patient sex.

We excluded a total of 3127 cancer cases that were not SCC, except in the cervix, for which we included both SCC and the HPV-attributable ACs according to the World Health Organization (WHO) 2020 definition [18].

Age-specific population size by sex and calendar year was obtained from the National Population Register. Data access was granted to address specific research aims, and the project data can be accessed on application to the CRN.

### HPV-Attributable Fraction

To estimate the number of cancer cases attributable to HPV, we multiplied the site-specific annual average number of cases with corresponding estimates of tumor HPV prevalence fractions (HPV-AF) from the scientific literature. Data on HPV-AF for noncervical cancers from Norwegian or Nordic studies were used, except for anal cancer where this was not available: 77% of oropharyngeal SCC [9], 100% of anal SCC [1], 47% of penile SCC [10], 35% of vulvar SCC [11], and 67% of vaginal SCC [8].

### Incidence Trends

We present age-standardized incidence rates per 100 000 person-years using the World Standard Population [19]. Joinpoint regression was used to evaluate the temporal incidence trends for each cancer type for both sexes based on the age-standardized incidence rates [20, 21]. Joinpoint regression finds the line giving the best fit to the data, where the line is allowed to be divided into multiple segments. To determine the optimal number of segments, the program starts with a single segment, and tests successively whether more segments give a better fit to the data. For each segment, as well as for the whole study period, an average annual percentage change (APC and AAPC, respectively) with 95% confidence interval (CI) was calculated.

Loess regression was used for prediction of future cancer incidence rates. We first fitted a (possibly) nonlinear regression line to the observed age-standardized incidence rates (1990–2023). The analysis was performed using the *loess* function in R, with the smoothing parameter alpha set equal to 1 [22]. Predictions of future incidence rates were performed by projecting the regression line into the period 2024–2038, obtained by applying the *predict* function to the loess regression object in R.

R code for the statistical analyses can be found here: github.com/staaln76/hpv-cancer-norway-trend.

## RESULTS

### Incidence Trends, 1990–2023

**Figure 1.**
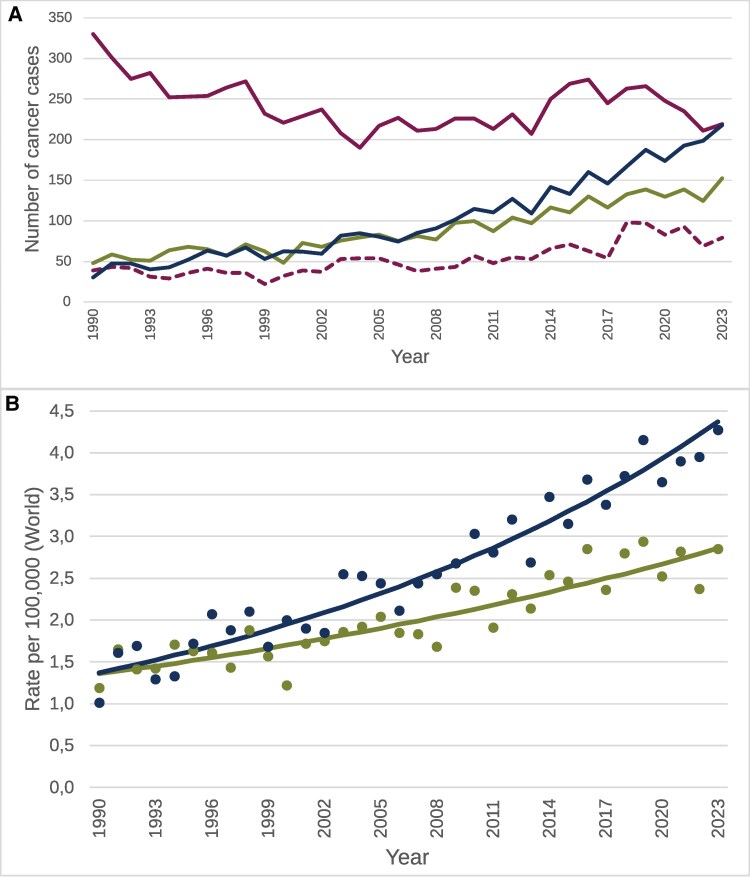
The figure is based on the following human papillomavirus (HPV)–attributable fractions (AF): cervix (squamous cell carcinoma [SCC] and adenocarcinoma [AC]), 100%; oropharynx (SCC), 77%; anus (SCC), 100%; vulva (SCC), 35%; vagina (SCC), 67%; penis (SCC), 47%. *A*, Number of cancer cases attributed to HPV from 1990 to 2023. Solid amber line: cervical SCC; dotted amber line: cervical AC; green line: noncervical HPV-attributable cancers in women (HPV-AF of SCC of oropharynx, anus, vulva, and vagina); blue line: HPV-attributable cancers in men (SCC of oropharynx, penis, and anus). *B*, Incidence trends for noncervical HPV-attributable cancers in Norway (1990–2023). Green: Noncervical HPV-attributable cancer in women (HPV-AF of SCC in oropharyngeal, anal, vulvar, and vaginal cancers); blue: HPV-attributable cancers in men (HPV-AF of SCC in oropharyngeal, penile, and anal cancers). Points denote observed age-standardized (world) incidence per 100 000 person-years. Lines denote the best-fitting trend line, and squares denote the joinpoints, from joinpoint regression analyses.

The total number of incident cancer HPV-attributable cases increased consistently throughout the study period (Table 1, Figure 1*A*). In 1990–1994, the average annual number of HPV-related cancer cases for both men and women was 474, but during the last 4 years (2020–2023), an average of 782 cases of oropharyngeal, anal, penile, vulvar, vaginal, and cervical SCCs in addition to cervical ACs occurred annually in Norway. In this period, HPV-related cancer types in women increased 20%, but for men the increase was 4.5-fold. In 2023, the total number for both sexes was 818 cancer cases in HPV-related tumor sites, which amounts to 2.2% of all cancer incidences in Norway in 2023 (2.9% in women and 1.5% in men) [23].

**Table 1.** Human Papillomavirus–Related Squamous Cell Carcinoma Incidence, by Sex, Cancer Site, and Time Period

Cancer Type	HPV-Related Cancer Incidence, Average Annual Cases (Attributed to HPV^a^)						
	Time Period						
	1990–1994	1995–1999	2000–2004	2005–2009	2010–2014	2015–2019	2020–2023
Women							
Total HPV-related cancers	414 (379)	396 (354)	371 (329)	397 (346)	436 (382)	531 (466)	512 (445)
Cervical (SCC)	288 (288)	255 (255)	217 (217)	219 (219)	225 (225)	263 (263)	228 (228)
Cervical (AC)	37 (37)	34 (34)	43 (43)	44 (44)	56 (56)	77 (77)	81 (81)
Oropharyngeal^b^	8 (6)	17 (13)	16 (12)	27 (21)	36 (28)	47 (36)	49 (38)
Anal	26 (26)	25 (25)	29 (29)	33 (33)	42 (42)	53 (53)	62 (62)
Vulvar	44 (16)	52 (18)	52 (18)	63 (22)	63 (22)	76 (27)	78 (27)
Vaginal	11 (7)	13 (9)	14 (9)	11 (7)	14 (9)	14 (10)	15 (10)
Men							
Total HPV-related cancers	60 (42)	82 (59)	99 (70)	122 (86)	165 (121)	219 (159)	270 (196)
Oropharyngeal^b^	29 (22)	45 (35)	59 (45)	73 (56)	111 (85)	146 (113)	185 (142)
Anal	9 (9)	12 (12)	11 (11)	14 (14)	18 (18)	23 (23)	25 (25)
Penile	23 (11)	24 (11)	29 (14)	35 (16)	36 (17)	50 (23)	61 (29)

Numbers are presented without digits. Rounding may therefore cause discrepancies in the total numbers.

Abbreviations: AC, adenocarcinoma; HPV, human papillomavirus; SCC, squamous cell carcinoma.

^a^Based on the following HPV-attributable fractions: cervix (SCC and AC), 100%; oropharynx (SCC), 77%; anus (SCC), 100%; vulva (SCC), 35%; vagina (SCC), 67%; penis (SCC), 47%.

^b^Oropharynx (SCC): base of tongue, tonsils, and oropharynx.

Even though CC is still the most common HPV-related cancer, HPV is now causing more cancers at other sites than the cervix (Table 1). By analyzing the combined incidence trend for HPV-attributable cancers other than CC (oropharyngeal, anal, penile, vulvar, and vaginal cancers), we demonstrated an annual increase of 2.8% (95% CI, 2.5%–3.1%; *P* < .01) in incidence rates over the 33-year period (Table 2). The incidence rate of HPV-attributable cancers in men (oropharyngeal, anal, and penile cancers) increased more rapidly than noncervical cancers in women (oropharyngeal, anal, vulvar, and vaginal cancer) (Figure 1*B*).

**Table 2.** Incidence Trend Analyses of All Human Papillomavirus–Attributable Cancers^a^ in Norway, 1990–2023

Organ	Histology	Sex	AAPC (95% CI)	Joinpoint	Subperiod	APC (95% CI)
Cervical	SCC	Women	−1.9 (−2.7 to −1.1)^b^	2004	1990–2004	−3.7 (−4.5 to −2.8)^c^
			…	2018	2004–2018	1.4 (0.4–2.4)^c^
			…	…	2018–2023	−6.0 (−9.9 to −1.9)^c^
	AC	Women	1.9 (.5–3.4)^b^	1999	1990–1999	−2.3 (−6.8 to 2.4)
				…	1999–2023	3.6 (2.4–4.7)^c^
	All	Women	−1.3 (−2.2 to −.4)^b^	2004	1990–2004	−2.9 (−3.8 to −2.0)^c^
			…	…	2004–2019	1.6 (0.7–2.5)^c^
			…	…	2019–2023	−6.3 (−11.9 to −0.3)^b^
Vulvar	SCC	Women	0.9 (0.2–1.6)^c^	None	…	…
Vaginal	SCC	Women	0.2 (−1.2 to 1.6)	None	…	…
Anal	SCC	Women	2.2 (1.5–2.9)^b^	None	…	…
	SCC	Men	1.8 (0.6–3.1)^c^	None	…	…
Oropharyngeal	SCC	Women	7.1 (4.5–9.8)^b^	1994	1990–1994	32.4 (8.4–61.7)^c^
				…	1994–2023	4.0 (3.0–5.1)^c^
	SCC	Men	4.5 (4.1–5.0)^b^	None	…	…
Penile	SCC	Men	1.7 (1.0–2.6)^b^	None	…	…
All noncervical HPV-related cancers^d^	SCC	Both	2.8 (2.5–3.1)^c^	None	…	…

Table shows joinpoint regression analyses.

Abbreviations: AAPC, average annual percentage change for the whole period (1990–2023); AC, adenocarcinoma; APC, annual percentage change for subperiod; CI, confidence interval; HPV, human papillomavirus; HPV-AF, human papillomavirus–attributable fraction; SCC, squamous cell carcinoma.

^a^Based on the following HPV-attributable fractions: cervix (SCC and AC), 100%; oropharynx (SCC), 77%; anus (SCC), 100%; vulva (SCC), 35%; vagina (SCC), 67%; penis (SCC), 47%.

^b^ *P* < .05.

^c^ *P* < .01.

^d^Men (penile SCC, anal SCC, oropharyngeal SCC) and women (anal SCC, vaginal SCC, vulvar SCC, oropharyngeal SCC).

**Figure 2.**
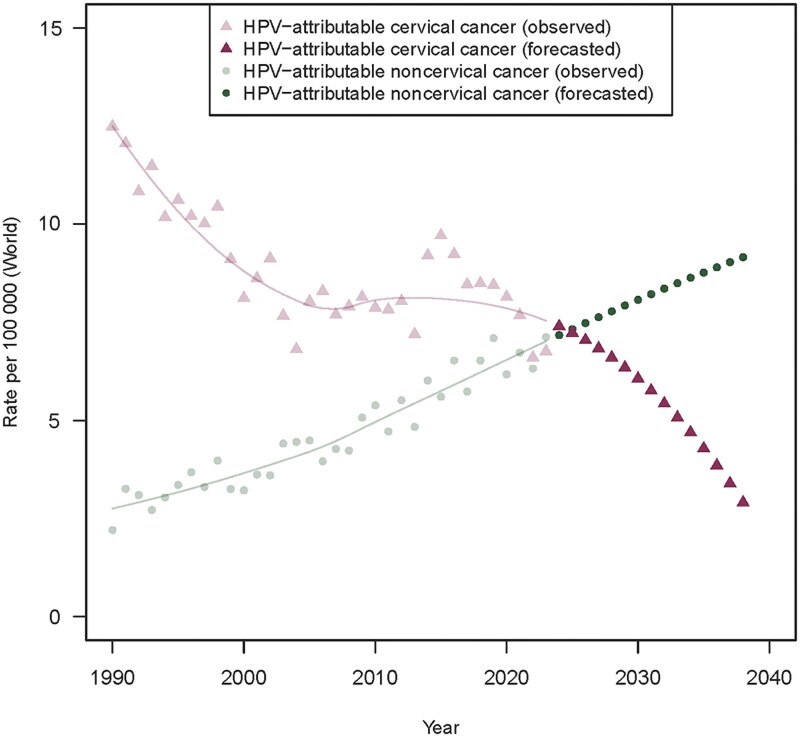
Forecast of human papillomavirus (HPV)–related cancer incidence trends in 2024–2038. Amber, cervical cancer (squamous cell carcinoma [SCC] and adenocarcinoma); green: noncervical HPV-attributable cancers in men and women (HPV-attributable fraction of SCC in vulvar, vaginal, anal, penile, and oropharyngeal cancers). Observed incidence trends (1990–2023) are indicated in soft colors.

The forecasted incidence trend for noncervical cancers is expected to continue increasing until 2038 (Figure 2). The incidence of CC (both SCC and ACs) started to decline by 6.3% annually in 2019 (95% CI, −11.9% to .3%; *P* < .05) and is expected to continue decreasing to 2038 (Figure 2).

**Figure 3.**
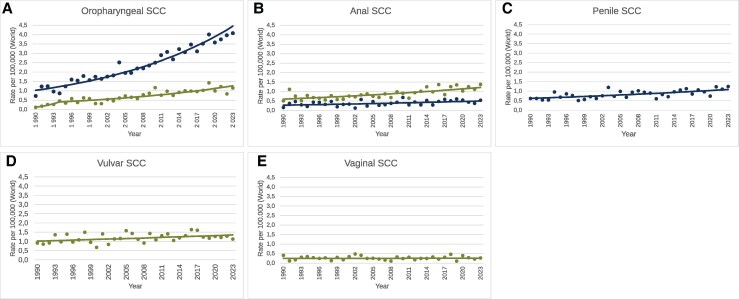
*A–E*, Incidence trends for human papillomavirus (HPV)–attributable cancer types among men (blue) and women (green) in Norway, 1990–2023. Points denote observed age-standardized (world) incidence per 100 000 person-years. Lines denote the best-fitting trend line and squares the joinpoints from joinpoint regression analyses. Abbreviation: SCC, squamous cell carcinoma.

The incidence of oropharyngeal SCC among men has increased more than the other HPV-attributable SCC studied. The overall incidence rate increased by 4.5% annually over the entire study period (95% CI, 4.1%–5.0%; *P* < .01) (Table 2, Figure 3*A*). Oropharyngeal SCC among women increased rapidly until 1994, followed by a more moderate increase until 2023. For the period 1994–2023, the increase was 4.0% annually (95% CI, 3.0%–5.1%; *P* < .01) (Table 2, Figure 3*A*).

Anal SCC incidence among men increased annually by 1.8% during the 33-year period (95% CI, .6%–3.1%; *P* < .05) (Table 2, Figure 3*B*). Anal SCC incidence among women increased annually by 2.2% (95% CI, 1.5%–2.9%; *P* < .01) for the whole 33-year period (Table 2, Figure 3*B*).

Penile SCC incidence was consistently increasing by 1.7% annually during 1990–2023 (95% CI, 1.0 to 2.6, *P* < .01) (Table 2, Figure 3*C*), vulvar SCC incidence was consistently increasing by 0.9% (95% CI, 0.2%–1.6%; *P* < .05) over the whole period 1990–2023 (Table 2, Figure 3*D*), and vaginal SCC was the rarest HPV-related cancer with no significant trend observed (Table 2, Figure 3*E*).

The analysis of the cervical SCC incidence trends for the period 1990–2023 revealed 3 distinct periods. From 1990 to 2004, cervical SCC decreased every year by 3.7% (95% CI, −4.5% to −2.8%; *P* < .01). From 2004, an annual increase of 1.4% was observed until 2018 (95% CI, 0.4% to 2.4%; *P* < .01). After 2018, a significant decrease of 6% was observed until 2023 (95% CI, −9.9% to −1.9%; *P* < .01). Overall, a significant average annual decrease in cervical SCC incidence of 1.9% was observed over the entire study period (95% CI, −2.7% to −1.1%) (Table 2). In contrast, cervical AC incidence did not change significantly until 1999. From 1999 until 2023, AC incidence followed an opposite direction to cervical SCC and increased significantly every year by 3.6% (95% CI, 2.4%–4.7%; *P* < .01) (Table 2).

Overall, CC (SCC and AC) has decreased by 6.3% annually since 2019 (95% CI, −11.9% to −0.3%; *P* < .05). The incidence trend for CC (SCC and AC) in 1990–2023 is shown in Figure 2 .

## DISCUSSION

This up-to-date report provides nationwide data on incidence trends for all HPV-attributable cancers in both men and women in Norway from 1990 to 2023. During this period, significant advancements have been made, including the implementation of the national HPV vaccination program for both boys and girls and the transition from cytology to HPV testing as the primary screening method for CC. These are crucial steps toward eliminating HPV-attributable diseases. However, only CC is prevented by screening and only younger generations are protected by HPV vaccination. Therefore, we also examine the projected incidence of the total burden of HPV-attributable diseases at the proposed CC elimination timepoint in Norway in 2038, if no extra preventive actions are made.

The landscape of HPV-related cancers in Norway is shifting. While cervical SCC incidence is decreasing, other HPV-attributable cancers are on the rise, with HPV causing more cancers at noncervical sites than cervix since 2022. This trend is particularly concerning for men, who face delayed HPV vaccination compared to women and a lack of screening.

Increased HPV exposure is a suggested reason for the observed rise in noncervical HPV-related cancers [24, 25], which fits with the HPV epidemics in high-income countries starting decades ago [5, 24]. Among Scandinavian women, there are secular trends of more partners and STIs [24 ]. The sexual repertoire is more varied than before, allowing transmission of HPV across more anatomic sites [26]. Since the dating apps gained popularity in Norway around 2012–2013, the incidence of chlamydia and gonorrhea has shown a rising trend up until today [27]. HPV is likely to follow the trend of other STIs, at least for age groups and HPV types not covered by HPV vaccination. However, in an HPV-vaccinated population, this trend is likely to turn.

We observed the greatest increase in HPV-attributable cancer incidence in oropharyngeal cancers. Although the increase was equally dramatic in women (4.0% annually after 1994) and men (4.5% annually), the burden in men observed in absolute numbers was 4 times higher. The proportion of cancers at this site attributable to HPV has dramatically increased in high-income countries [28]. For both oropharyngeal and penile cancer, previous studies indicate that it is the HPV-attributable cancers that are increasing, and that the number of cases caused by other risk factors are steady [9, 10]. In the present study, only oropharyngeal and penile SCCs are increasing, while non-SCCs are stable. Incidence rates among those <55 years of age appear to be stabilizing [29], suggesting that major shifts in sexual behavior may have occurred before 1990. However, as the natural history of oropharyngeal cancer is not well established, this is, for now, speculative. The rising rates of other STIs in Norway, such as chlamydia and gonorrhea [27], suggest a potential increase in risky sexual behaviors. The long-term impact of this remains to be seen.

However, tobacco and alcohol use have historically been the primary drivers of head and neck cancers. To fully understand the observed increase, we need to disentangle not only the changes in exposure to HPV infection but also other risk factors [30]. Tobacco and alcohol consumption patterns are likely to vary across birth cohorts. For example, the rise oropharyngeal cancers among women until 1994 likely reflects factors other than HPV. In Norway, male smoking rates increased until the 1950s, and female rates until the 1970s. Since then, smoking prevalence has declined in all cohorts [31]. Along with this trend, we have observed a significant decrease in tobacco-related lung cancer rates in Norway for both men and women. Alcohol is also a risk factor for cancer in general, and among the HPV-related diseases, oropharyngeal cancer especially [30]. Alcohol consumption in Norway has increased from 1990 to 2008; however, it has stabilized or even declined since then, especially among the young [32].

The general increase in noncervical HPV-related cancers is not a Norwegian-specific phenomenon. The trend is also seen in other countries [6, 33, 34]. In the United States, the incidence of HPV-related oropharyngeal cancers has surpassed CC [28].

The accuracy, completeness, timeliness, and comparability of data at the Cancer Registry of Norway have been meticulously documented over time. Therefore, the commonly suggested explanation of improvements in coding routines and changes in histological verification of cancers is unlikely to account for the increasing trends of noncervical HPV-attributable cancers. Furthermore, to our knowledge there have not been any major changes in diagnostics for these cancers and there are no available screening programs. Therefore, our findings are in line with the hypothesis that increased exposure and an increase of HPV prevalence in the population play the main role. Early sexual debut and more sexual partners as well as previous STIs are well-known risk factors of HPV-attributable cancers [35]. The time interval from infection to noncervical cancer fits well with the changes in sexual behaviors over the past decades [36].

While the coronavirus disease 2019 pandemic may have impacted cancer detection, we believe it did not significantly affect CC incidence in Norway. The screening program remained largely unaffected, with consistent sample collection and conization rates [37]. Moreover, if underdiagnosis occurred, we would expect an increase in incidence in subsequent years, which is not observed.

The forecasted incidences for 2024 to 2038 were based on projecting the incidence trends observed until 2023 and 14 years into the future. This approach rests on the assumption that no new factors will impact the incidence rates in the near future. HPV vaccination has caused a rapid decline in vaccine-targeted types in young, highly vaccinated birth cohorts, a profound impact not yet mirrored in older, predominantly unvaccinated populations [38]. Herd immunity in younger generations is expected to eventually lead to a decrease in HPV circulation in older age groups over time. Also, due to the long time from HPV infection to cancer, especially for noncervical cancers, it may take decades for a decrease in HPV in the population to manifest in the form of reduced cancer incidences. For noncervical cancers, HPV-vaccinated individuals are not reaching cancer ages in the next few decades, implying that the observed increasing incidence trend, although slightly starting to flatten out, would continue until 2038. For CC, vaccinated individuals have already started to reach cancer-prone ages and may be one of the causes of the decreasing trend seen from 2018. By projecting the observed incidence rates into the period 2024–2038, we obtained a decrease in incidence rates, reflecting that the vaccinated cohorts came closer to the peak CC incidence age. Here, we included both SCC and AC of the cervix, as both vaccination and HPV testing targets both types, as opposed to historical cytology screening alone. A more comprehensive model-based forecast of cancer incidence rates was outside the scope of the study. We note, however, that our forecast was very much in line with previous findings by Portnoy et al [39], with predicted elimination of CC in Norway by 2038 using the WHO definition of 4 cases per 100 000.

CC screening has significantly reduced cervical SCC incidence in Norway. Cytology screening halved CC cases from 1961–2010 [40]. The decline has been explained by the prevention of SCC. In our study, we observed an initial decrease in incidence until 2014, followed by a slight increase until 2018, and then a subsequent 6% annual decrease leading to 2023. However, cervical AC incidence, which is harder to detect and prevent by cytology screening [41], has steadily increased since the 1950s. The shift from primary cytology to HPV testing in screening, with higher sensitivity for precancers, may temporarily have led to increased detection of precancer and cancer cases, before a higher secondary prevention was achieved [25, 42]. This level seems to be reached for cervical SCC in 2018 when the incidence trend turned. We expect AC to follow the same trend within a reasonable time.

The recent decline in incidence rates for CC in women <30 years of age might be the very beginning of the HPV vaccination effect among women in Norway. Other Nordic countries have already observed a decrease in CC incidence rates [29, 43]. In addition to sexual behavior patterns, these countries are very similar in prevaccination exposure to HR-HPV, cervical screening methodology, and childhood vaccination programs. However, the differences in timing and level of catch-up vaccination between the countries might explain the variation in current CC incident rates [43]. As more HPV-vaccinated cohorts from the childhood vaccination program reach precancer and cancer ages in Norway, CC rates should continue to decline.

Screening programs are unavailable for HPV-related cancers beyond CC, despite the existence of precursors and detection methods for these lesions in the vulva, vagina, penis, and anus [35, 44, 45]. For oropharyngeal cancers, other potential screening methods, such as detection of HPV-16 E6 antibodies, are considered [46]. Noncervical HPV-attributable cancers often develop later in life, delaying the impact of vaccination. Women may benefit from gynecological examinations during CC screening and might explain why women have a lesser increase in noncervical cancers during the study period. The impact of vaccination on noncervical cancers may also have started to show [47, 48]. Men, who began vaccination later and lack relevant screening programs, are expected to have a continued increases in HPV-attributable cancers. Our analysis confirms that the total burden of noncervical HPV-attributable cancers surpassed CC in 2021, accounting for population growth. This is in line with previous findings [5].

The major strength of the present study is the accuracy and scale of the cancer data presented. Reporting of cancer is mandatory by law in Norway, and cancer registration is thus considered virtually complete [17]. However, this study has limitations due to uncertainties in HPV-AFs for each cancer. Since HPV-AFs vary over time [10, 11], our use of constant AFs may over- or underestimate HPV-attributable cancer incidences for certain time periods. Precise estimates of rates of HPV-attributable cancer incidences will require HPV genotyping of all incident cancer cases, but this is not current practice for HPV-related cancers in Norway.

Furthermore, this is an observational study and does not reveal explanations for the incidence trends. This applies especially to noncervical cancers, which are relatively rare and may have multifactorial etiology and important risk factors additional to HPV. Changes in detection, diagnosis, reporting, and coding practices influence cancer trends, and we cannot rule out that these are contributing to the observed increase. However, the trends observed in this study are consistent over time and across different diagnoses without any abrupt changes. Such findings would not be expected if these were the major factors.

In summary, while CC moves toward elimination, due to screening and HPV vaccination, other HPV-attributable cancers are on the rise. Among these, oropharyngeal cancers in men now have the highest increase in incidence rates. This trend is expected to continue, and therefore it is important that the total burden of HPV-attributable disease is not put in the shadow of the successful prevention of CC.

This study underscores the need for a broader perspective in the fight against HPV-attributable cancers and that this is not solely a women's health issue. Our findings strongly support the adoption of more aggressive strategies for HPV-attributable cancer reduction in both men and women, such as expanding the vaccination program to include unvaccinated men and high-risk groups as well as targeted screening and increased awareness. Screening options for noncervical cancers should be continued to be explored, like screening for HPV antibodies demonstrated to be sensitive and specific for oropharyngeal cancer in particular and that have been detected in blood several years before cancer incidence [46]. However, such screening will not help prevent cancer before treatment options for precancerous lesions are available also for noncervical HPV-attributable cancers [49].

## Note

***Financial support.*** There is no financial support provided for this study.
